# Supplementary figures and images for: Aberrant methylation and expression of TNXB promote chondrocyte apoptosis and extracullar matrix degradation in hemophilic arthropathy via AKT signaling
Source: eLife. 2024 May 31;13:RP93087. doi: 10.7554/eLife.93087 (PMC11142640; doi:10.7554/eLife.93087)

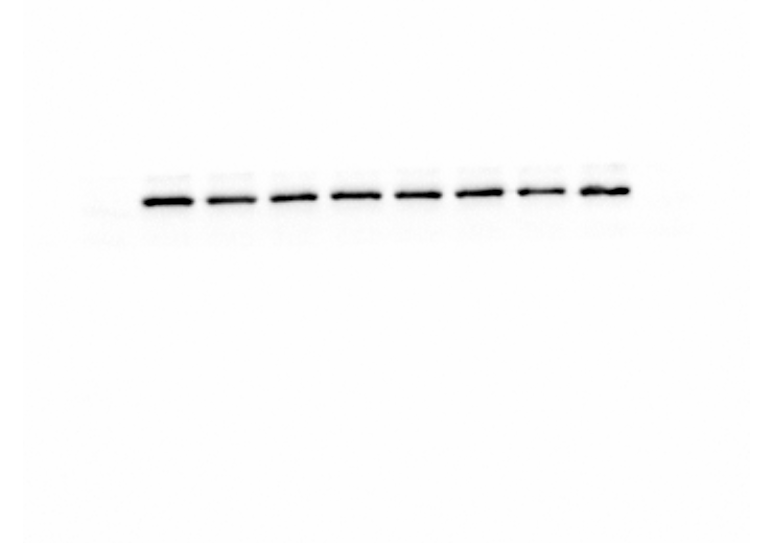

Supplement: Figure 4—source data 1. [file elife-93087-fig4-data1.zip › Original file for the Western blot analysis of β-actin in Figure 4B/Figure 4B_β-actin_sourceblot.tif]

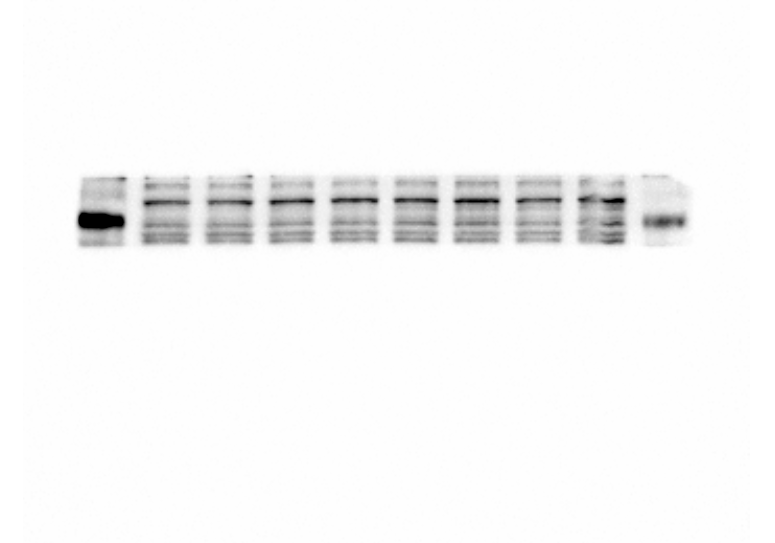

Supplement: Figure 4—source data 2. [file elife-93087-fig4-data2.zip › Original file for the Western blot analysis of Tnxb in Figure 4B/Figure 4B_Tnxb_sourceblot.tif]

**B)**

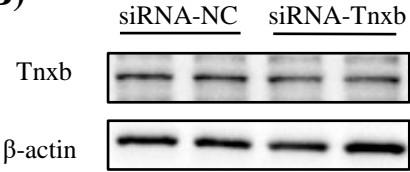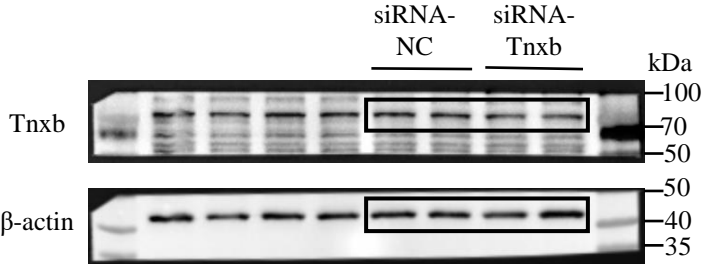

Supplement: Figure 4—source data 3. [file elife-93087-fig4-data3.pdf]

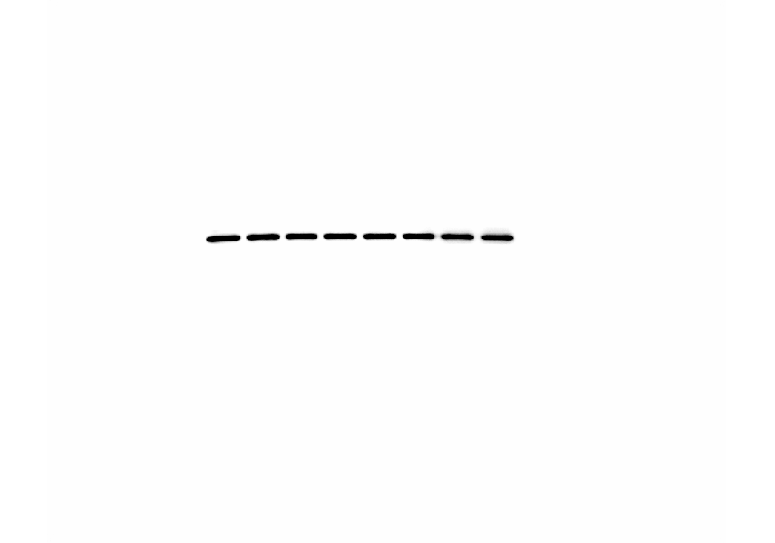

Supplement: Figure 4—source data 4. [file elife-93087-fig4-data4.zip › Original file for the Western blot analysis of β-actin in Figure 4E/Figure 4E_β-actin_sourceblot.tif]

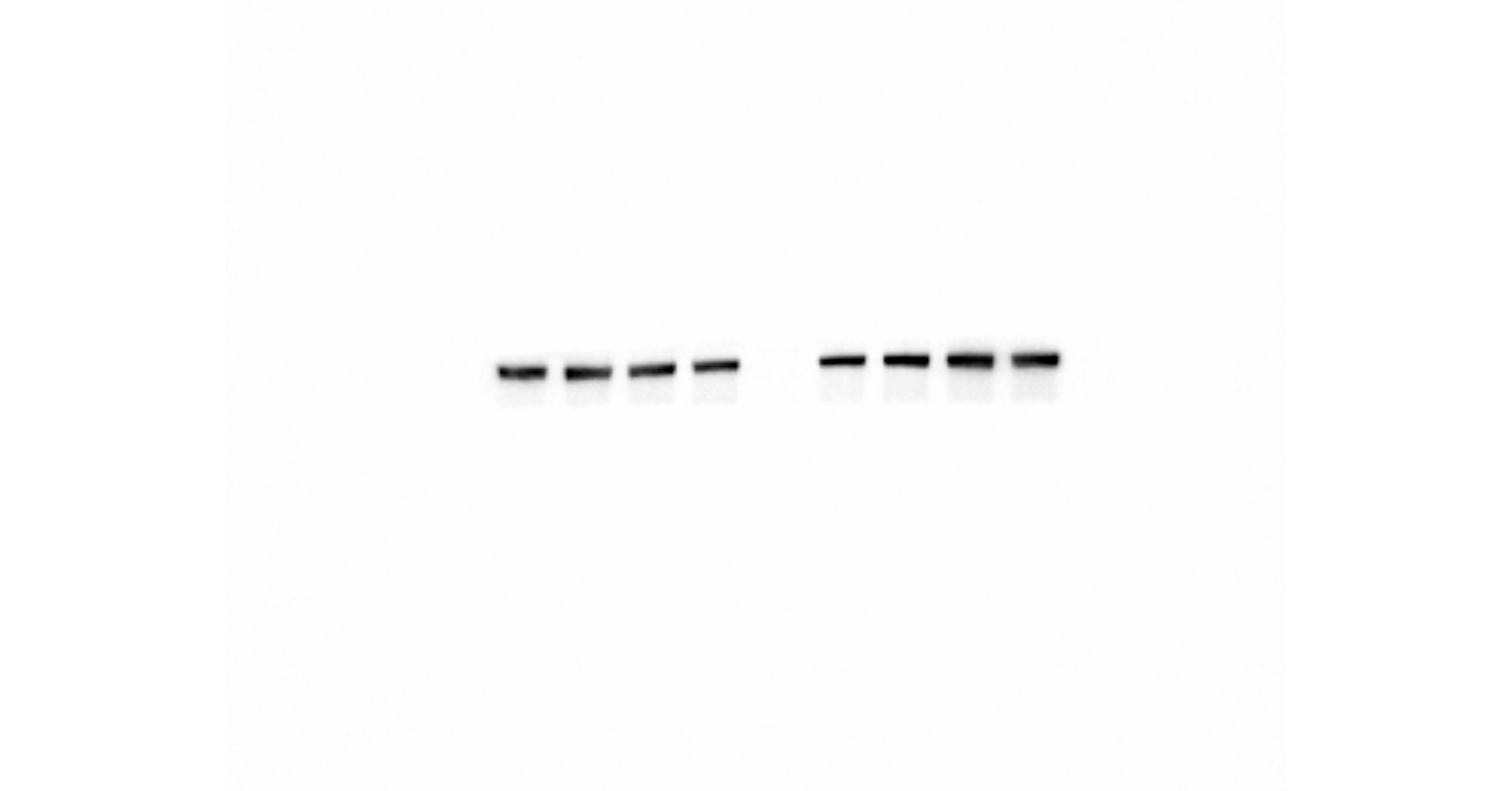

Supplement: Figure 4—source data 5. [file elife-93087-fig4-data5.zip › Original file for the Western blot analysis of Col2a1 in Figure 4E/Figure 4E_Col2a1_sourceblot.tif]

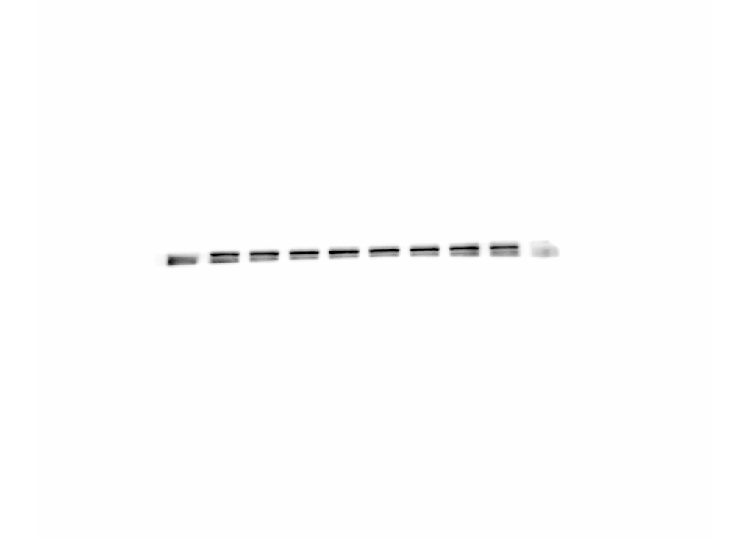

Supplement: Figure 4—source data 6. [file elife-93087-fig4-data6.zip › Original file for the Western blot analysis of Mmp13 in Figure 4E/Figure 4E_Mmp13_sourceblot.tif]

E)

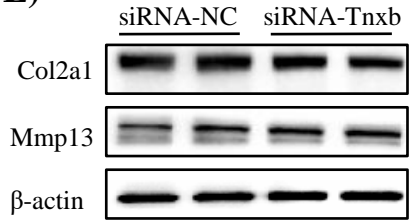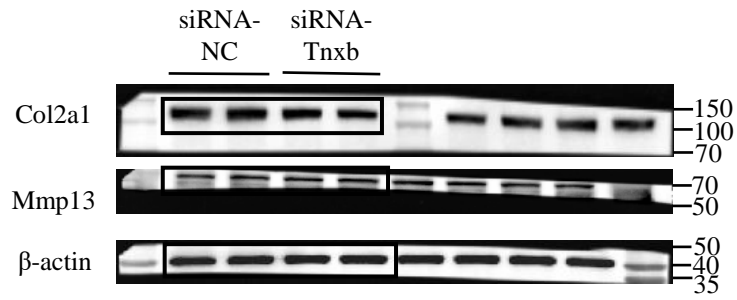

Supplement: Figure 4—source data 7. [file elife-93087-fig4-data7.pdf]

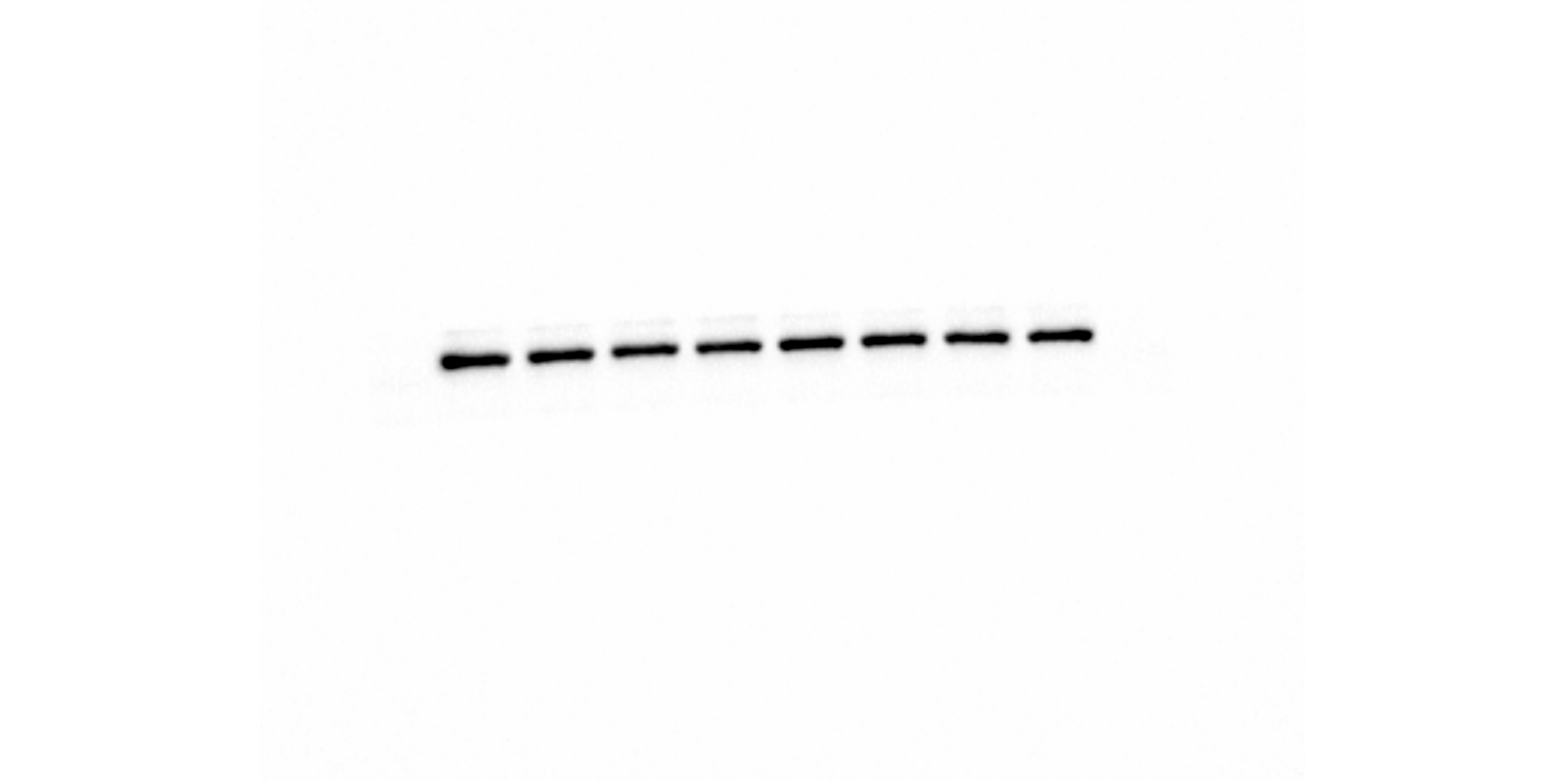

Supplement: Figure 5—source data 1. [file elife-93087-fig5-data1.zip › Original file for the Western blot analysis of β-actin in Figure 5C/Figure 5C_β-actin_sourceblot.tif]

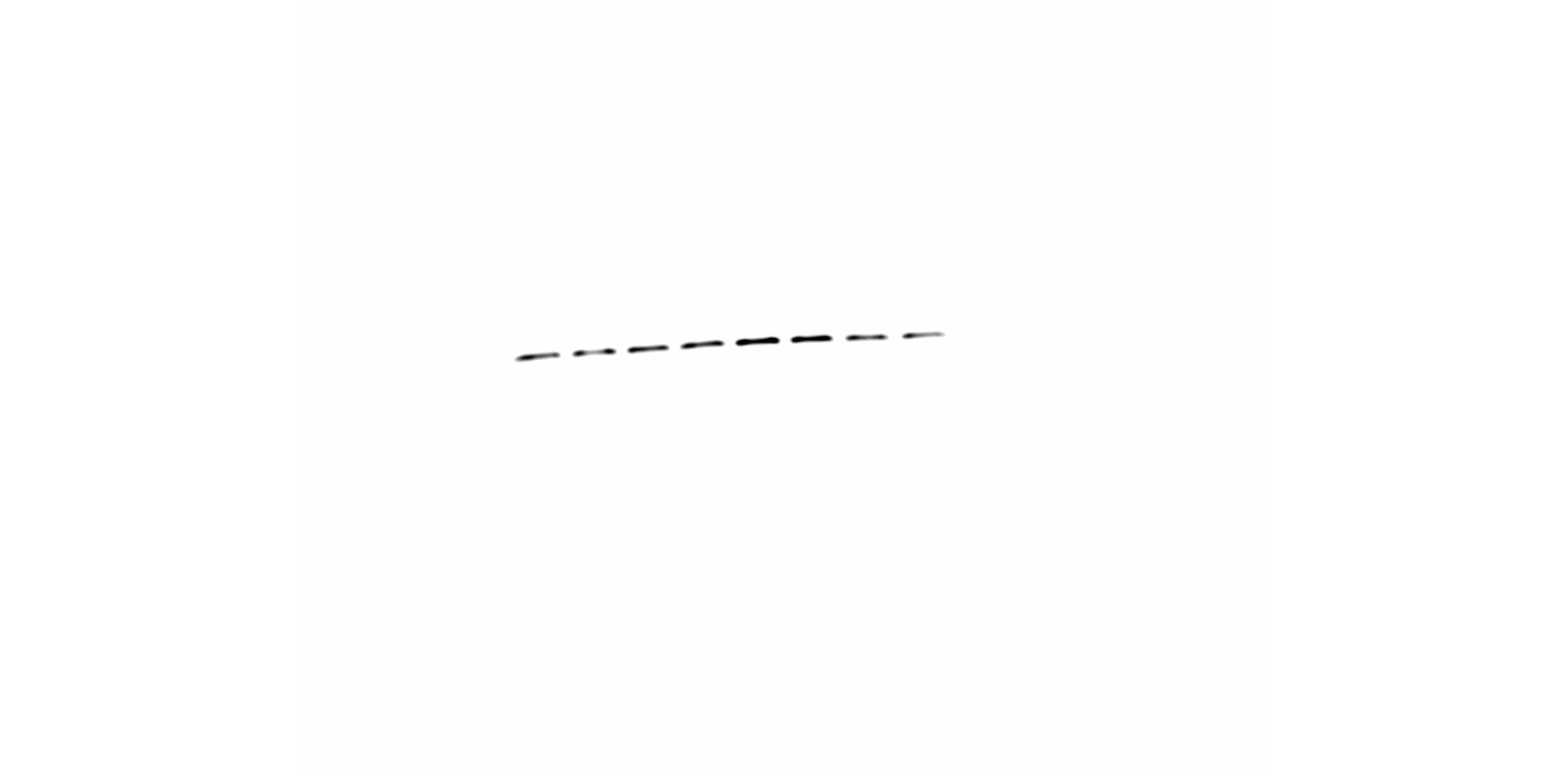

Supplement: Figure 5—source data 2. [file elife-93087-fig5-data2.zip › Original file for the Western blot analysis of Bax in Figure 5C/Figure 5C_Bax_sourceblot.tif]

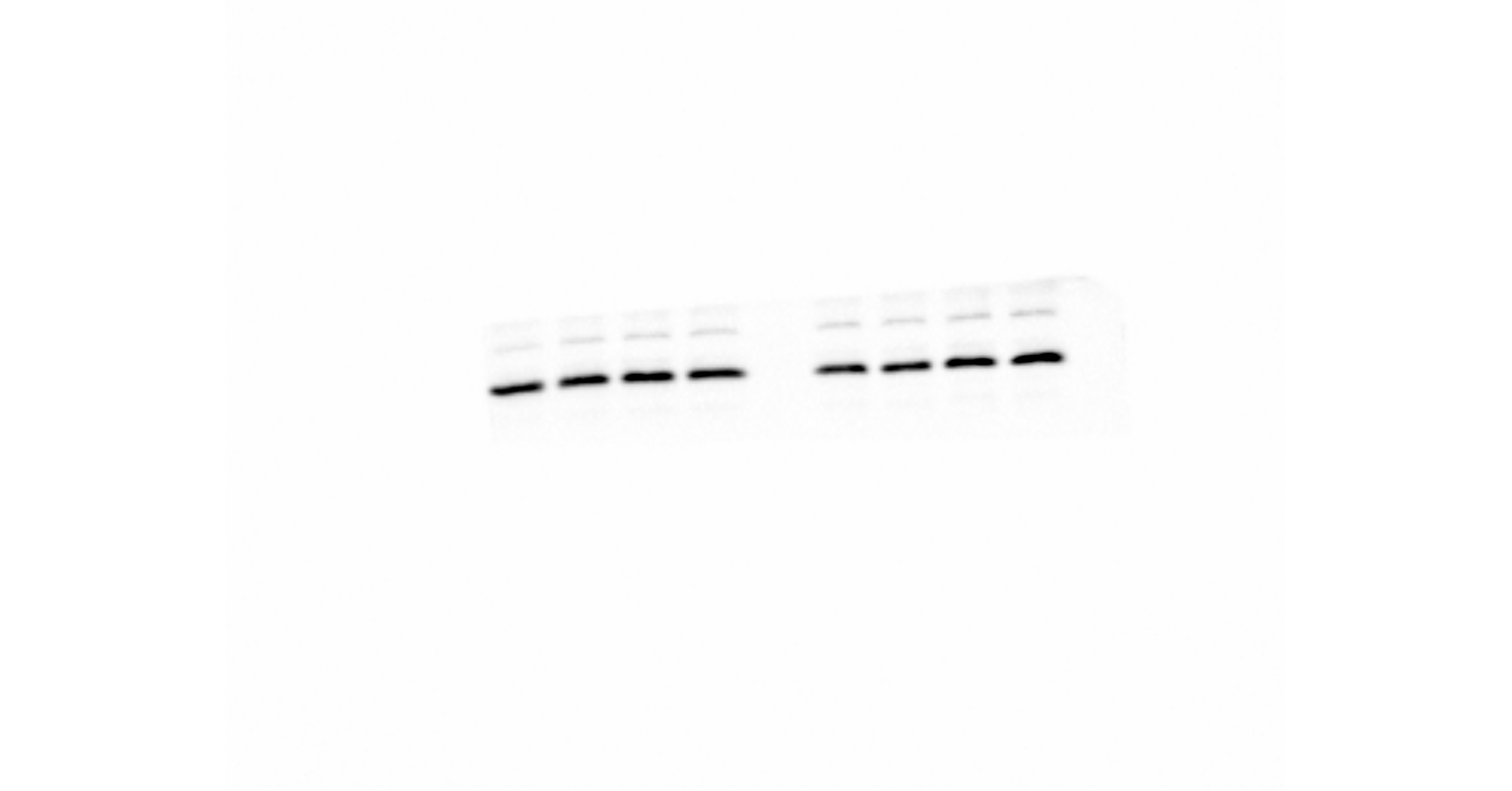

Supplement: Figure 5—source data 3. [file elife-93087-fig5-data3.zip › Original file for the Western blot analysis of Bcl-2 in Figure 5C/Figure 5C_Bcl-2_sourceblot.tif]

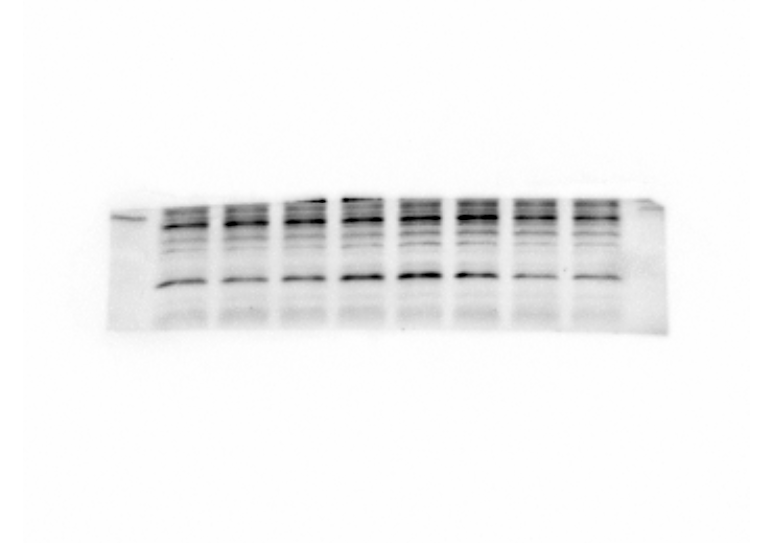

Supplement: Figure 5—source data 4. [file elife-93087-fig5-data4.zip › Original file for the Western blot analysis of cleaved-caspase3 in Figure 5C/Figure 5C_cleaved-caspase3_sourceblot.tif]

C)

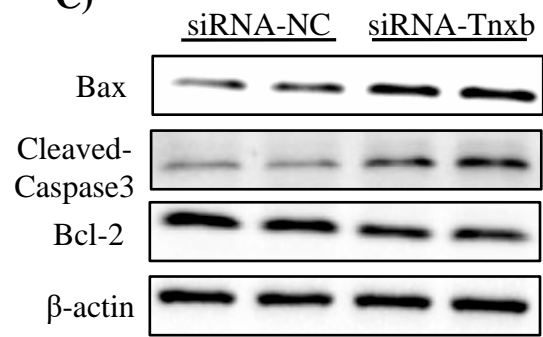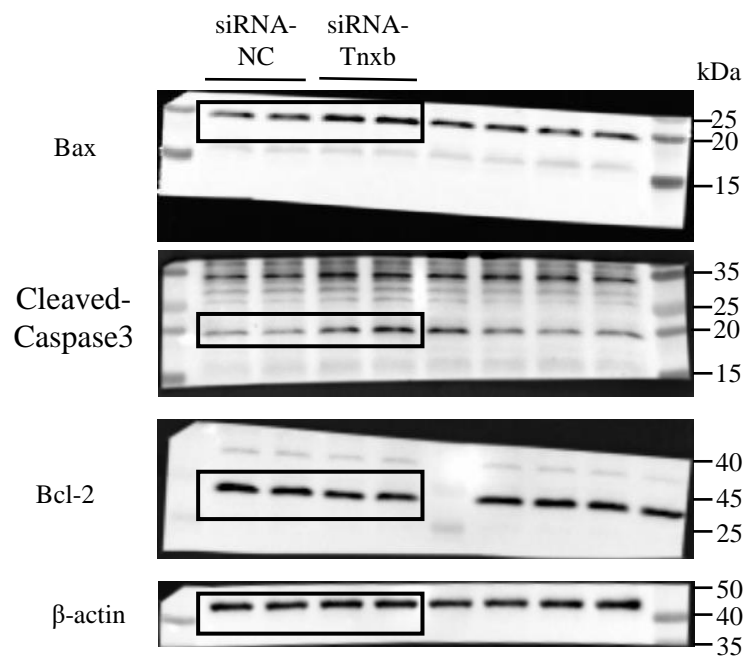

Supplement: Figure 5—source data 5. [file elife-93087-fig5-data5.pdf]

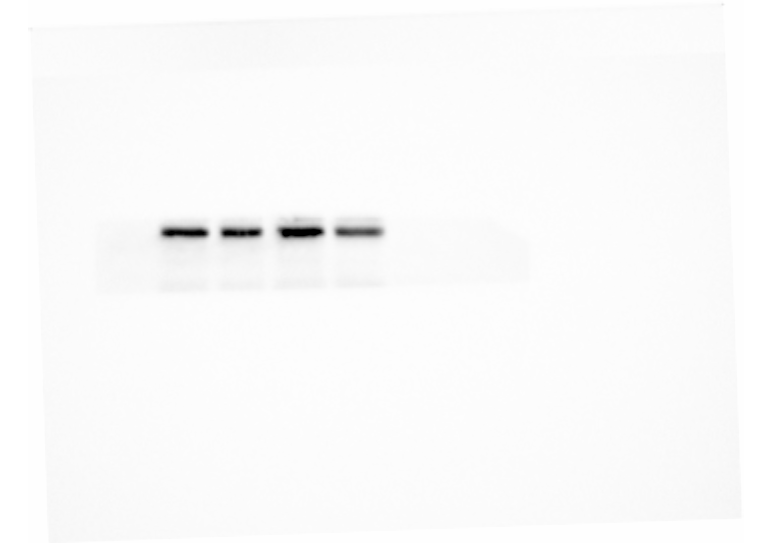

Supplement: Figure 6—source data 1. [file elife-93087-fig6-data1.zip › Original file for the Western blot analysis of β-actin in Figure 6A/Figure 6A_β-actin_sourceblot.tif]

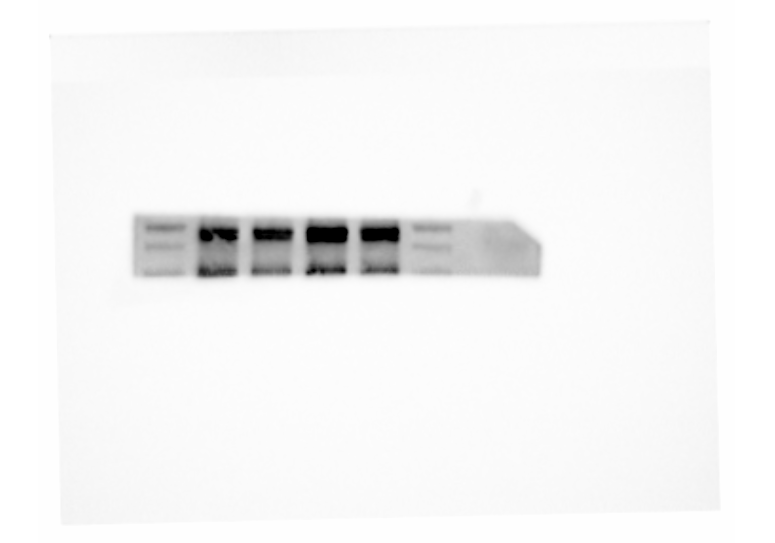

Supplement: Figure 6—source data 2. [file elife-93087-fig6-data2.zip › Original file for the Western blot analysis of Col2a1 in Figure 6A/Figure 6A_Col2a1_sourceblot.tif]

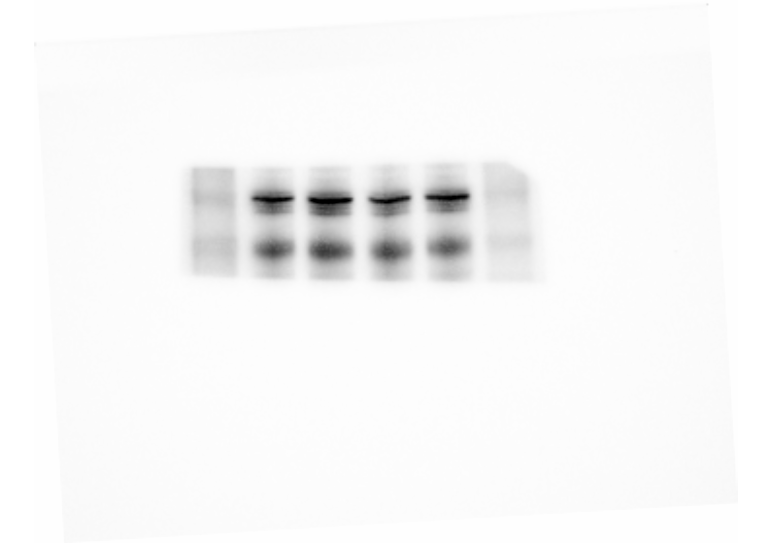

Supplement: Figure 6—source data 3. [file elife-93087-fig6-data3.zip › Original file for the Western blot analysis of Mmp13 in Figure 6A/Figure 6A_Mmp13_sourceblot.tif]

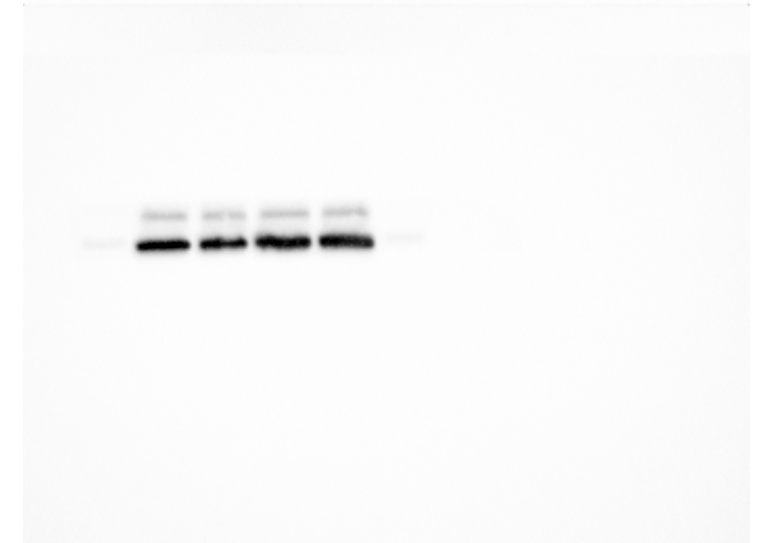

Supplement: Figure 6—source data 4. [file elife-93087-fig6-data4.zip › Original file for the Western blot analysis of AKT1 in Figure 6A/Figure 6A_AKT1_sourceblot.tif]

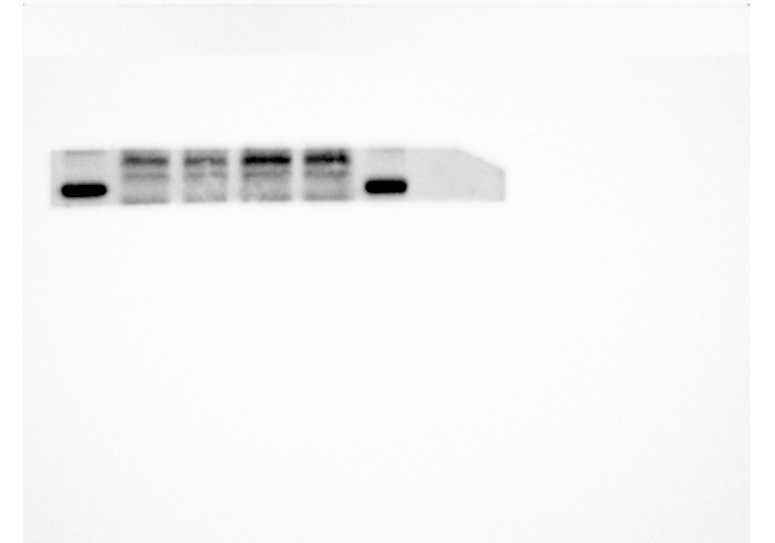

Supplement: Figure 6—source data 5. [file elife-93087-fig6-data5.zip › Original file for the Western blot analysis of pAKT1 in Figure 6A/Figure 6A_pAKT1_sourceblot.tif]

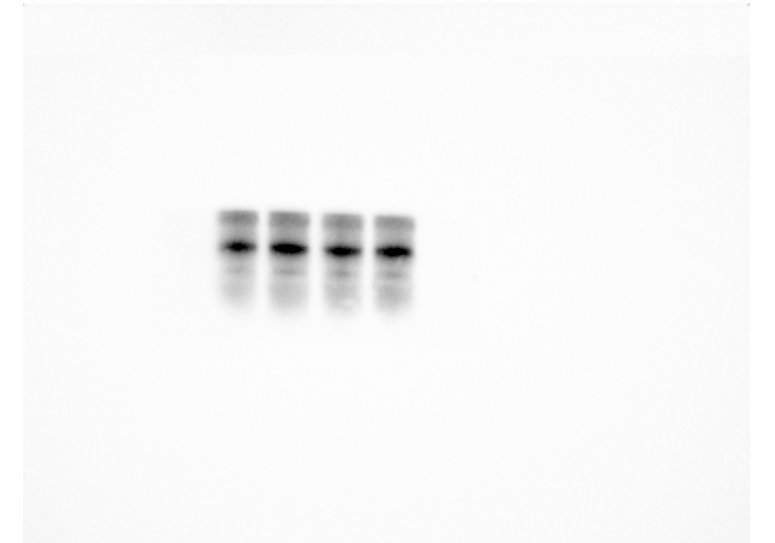

Supplement: Figure 6—source data 6. [file elife-93087-fig6-data6.zip › Original file for the Western blot analysis of Bax in Figure 6A/Figure 6A_Bax_sourceblot.tif]

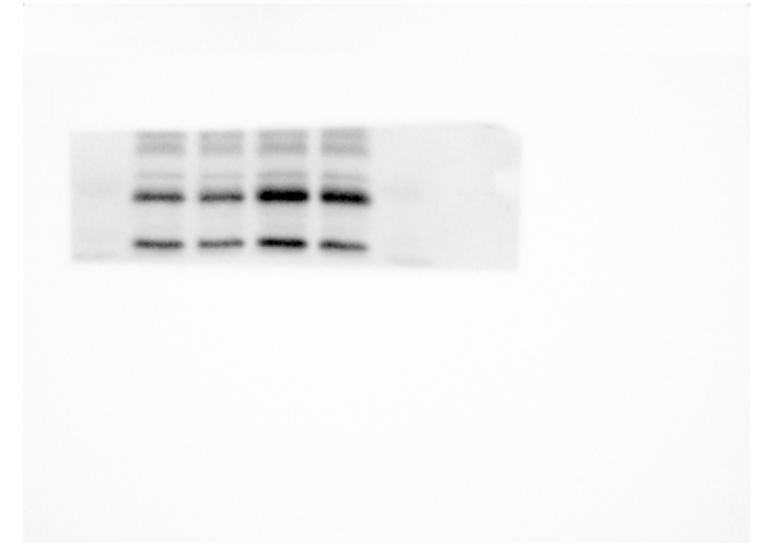

Supplement: Figure 6—source data 7. [file elife-93087-fig6-data7.zip › Original file for the Western blot analysis of Bcl-2 in Figure 6A/Figure 6A_Bcl-2_sourceblot.tif]

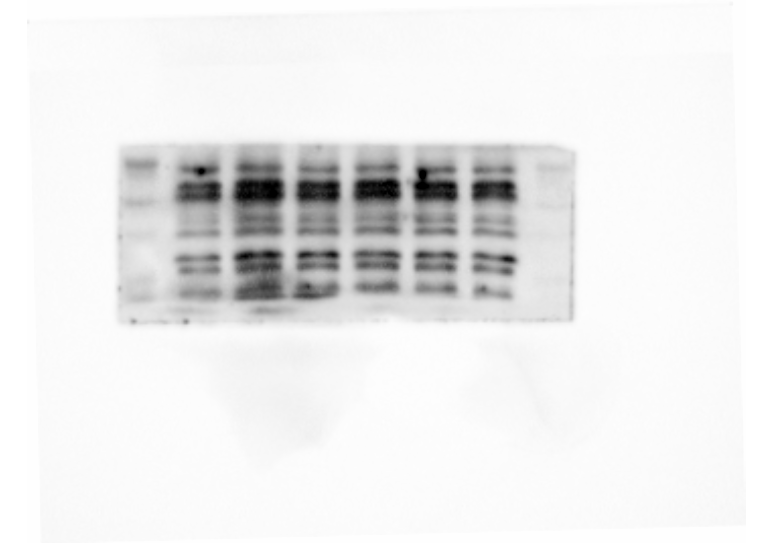

Supplement: Figure 6—source data 8. [file elife-93087-fig6-data8.zip › Original file for the Western blot analysis of cleaved-caspase9 in Figure 6A/Figure 6A_cleaved-caspase9_sourceblot.tif]

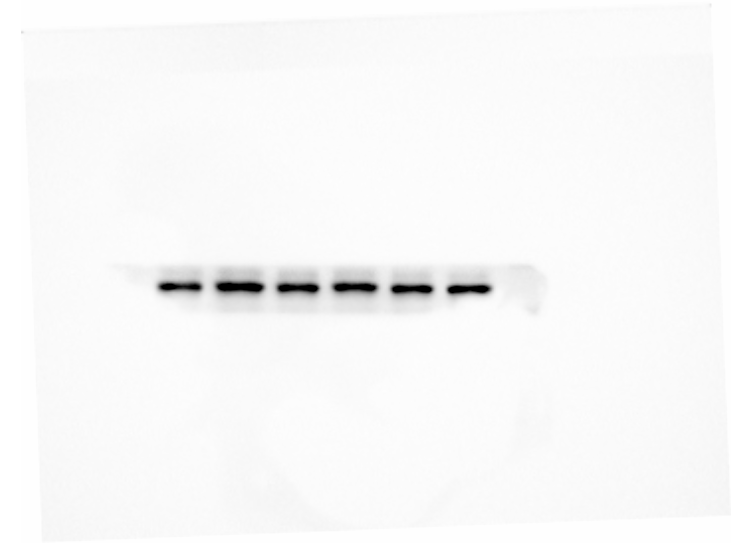

Supplement: Figure 6—source data 9. [file elife-93087-fig6-data9.zip › Original file for the Western blot analysis of cleaved-caspase9-β-actin in Figure 6A/Figure 6A_cleaved-caspase9-β-actin_sourceblot.tif]

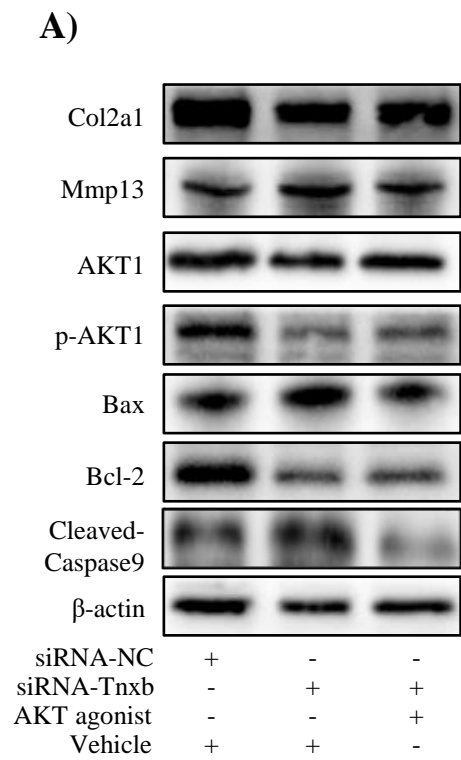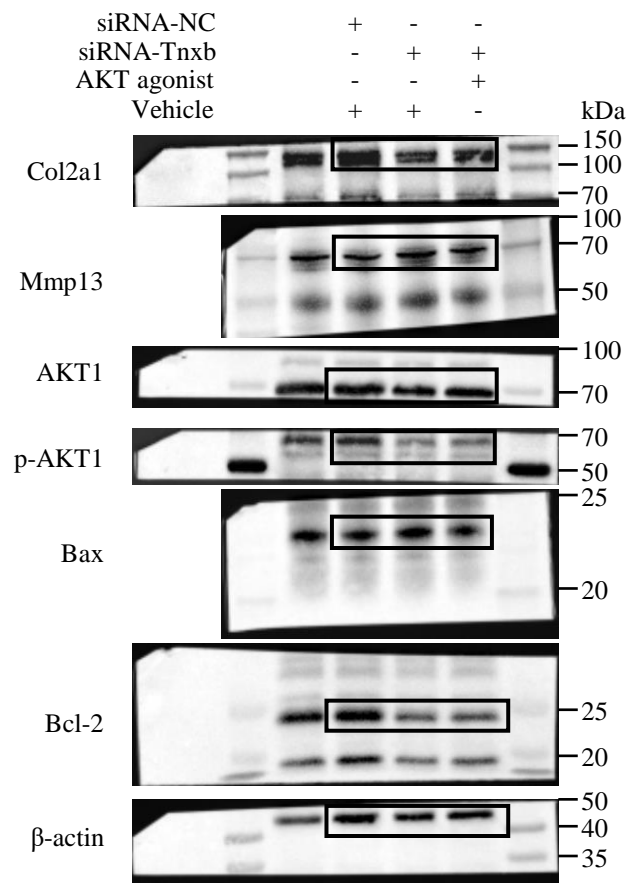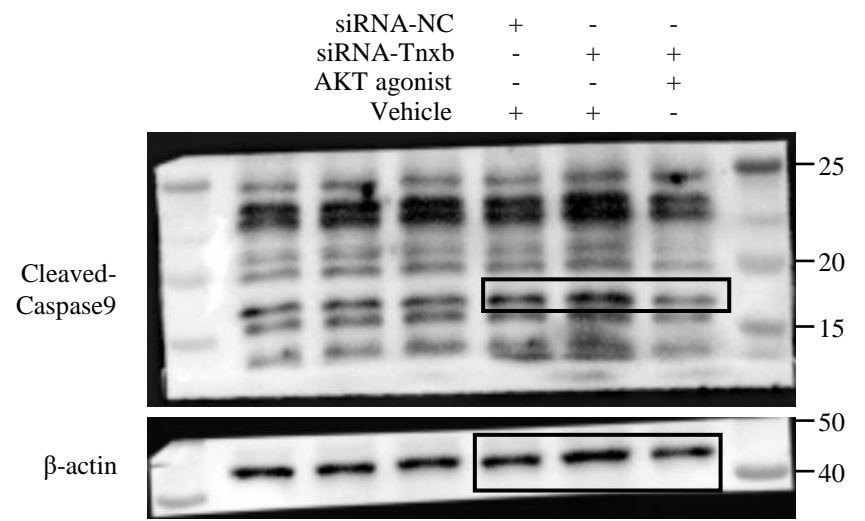

Supplement: Figure 6—source data 10. [file elife-93087-fig6-data10.pdf]

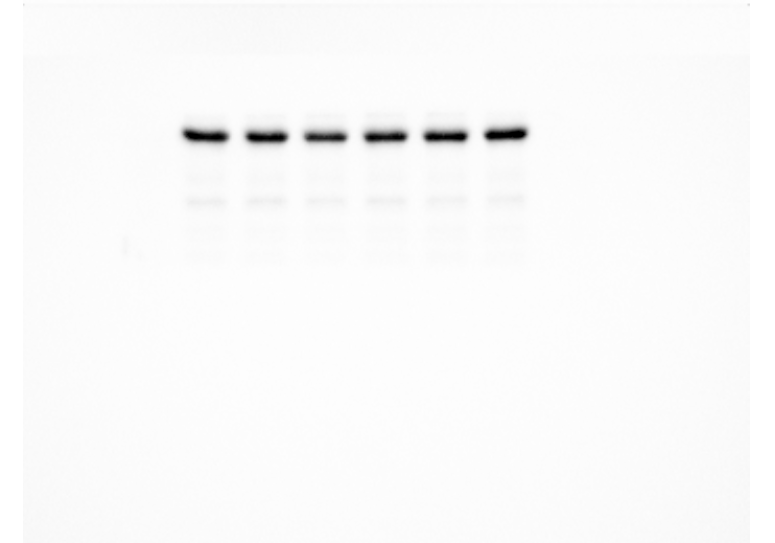

Supplement: Figure 6—figure supplement 1—source data 1. [file elife-93087-fig6-figsupp1-data1.zip › Original file for the Western blot analysis of β-actin in Figure 6-Figure supplements 1/Figure 6-Figure supplement 1A_β-actin_sourceblot.tif]

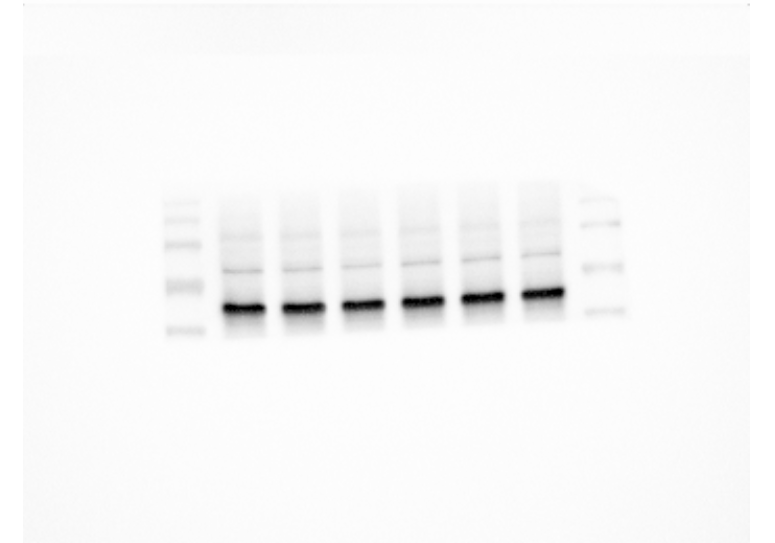

Supplement: Figure 6—figure supplement 1—source data 2. [file elife-93087-fig6-figsupp1-data2.zip › Original file for the Western blot analysis of AKT1 in Figure 6-Figure supplements 1/Figure 6-Figure supplement 1A_AKT1_sourceblot.tif]

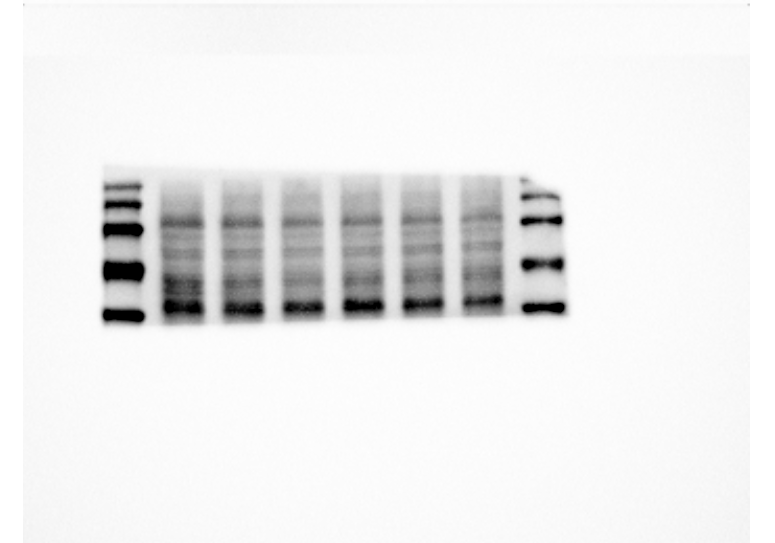

Supplement: Figure 6—figure supplement 1—source data 3. [file elife-93087-fig6-figsupp1-data3.zip › Original file for the Western blot analysis of pAKT1 in Figure 6-Figure supplements 1/Figure 6-Figure supplement 1A_pAKT1_sourceblot.tif]

A)

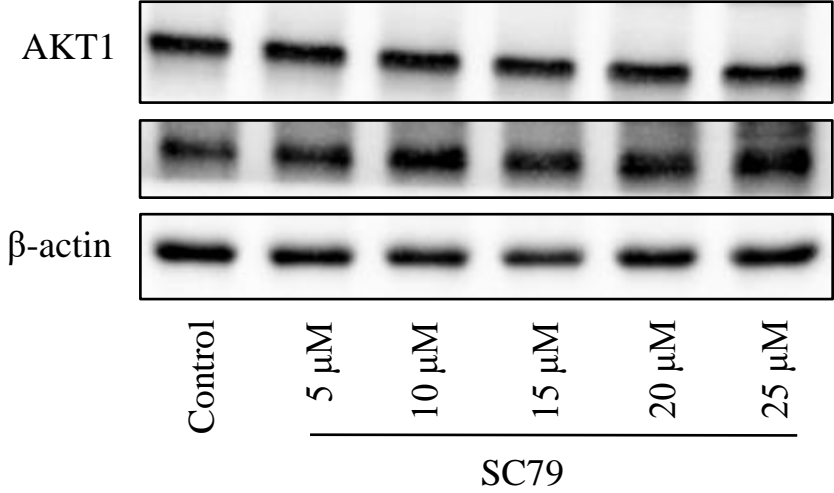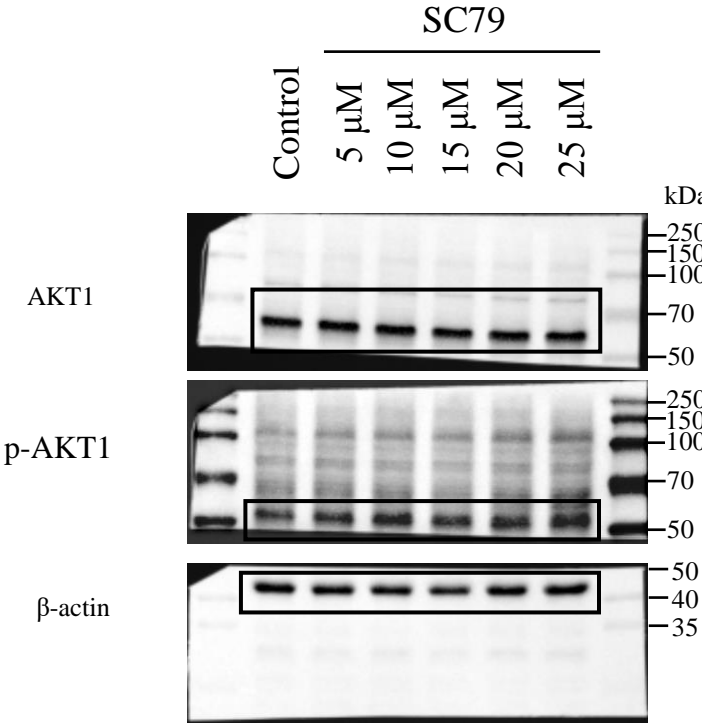

Supplement: Figure 6—figure supplement 1—source data 4. [file elife-93087-fig6-figsupp1-data4.pdf]

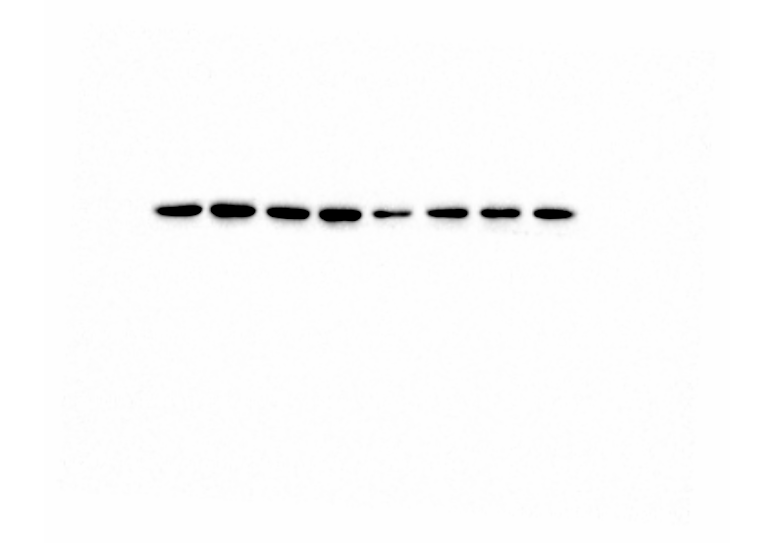

Supplement: Figure 6—figure supplement 2—source data 1. [file elife-93087-fig6-figsupp2-data1.zip › Original file for the Western blot analysis of β-actin in Figure 6-Figure supplements 1/Figure supplement 7A_β-actin_sourceblot.tif]

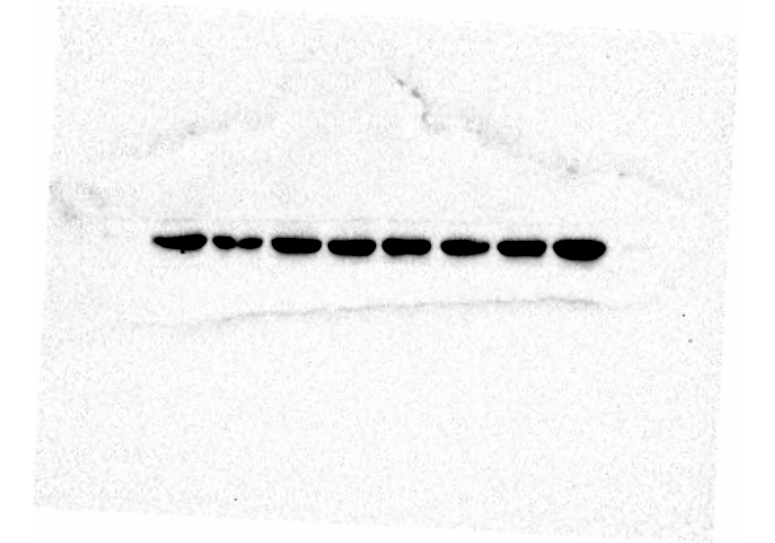

Supplement: Figure 6—figure supplement 2—source data 2. [file elife-93087-fig6-figsupp2-data2.zip › Original file for the Western blot analysis of pSmad2 in Figure 6-Figure supplements 1/Figure supplement 7A_pSmad2_sourceblot.tif]
